# Bottlenecks and the Maintenance of Minor Genotypes during the Life Cycle of *Trypanosoma brucei*

**DOI:** 10.1371/journal.ppat.1001023

**Published:** 2010-07-29

**Authors:** Michael Oberle, Oliver Balmer, Reto Brun, Isabel Roditi

**Affiliations:** 1 Swiss Tropical and Public Health Institute, Basel, Switzerland; 2 Institut für Zellbiologie, Universität Bern, Bern, Switzerland; University of Georgia, United States of America

## Abstract

African trypanosomes are digenetic parasites that undergo part of their developmental cycle in mammals and part in tsetse flies. We established a novel technique to monitor the population dynamics of *Trypanosoma brucei* throughout its life cycle while minimising the confounding factors of strain differences or variation in fitness. Clones derived from a single trypanosome were tagged with short synthetic DNA sequences in a non-transcribed region of the genome. Infections were initiated with mixtures of tagged parasites and a combination of polymerase chain reaction and deep sequencing were used to monitor the composition of populations throughout the life cycle. This revealed that a minimum of several hundred parasites survived transmission from a tsetse fly to a mouse, or vice versa, and contributed to the infection in the new host. In contrast, the parasites experienced a pronounced bottleneck during differentiation and migration from the midgut to the salivary glands of tsetse. In two cases a single tag accounted for ≥99% of the population in the glands, although minor tags could be also detected. Minor tags were transmitted to mice together with the dominant tag(s), persisted during a chronic infection, and survived transmission to a new insect host. An important outcome of the bottleneck within the tsetse is that rare variants can be amplified in individual flies and disseminated by them. This is compatible with the epidemic population structure of *T. brucei*, in which clonal expansion of a few genotypes in a region occurs against a background of frequent recombination between strains.

## Author Summary

African trypanosomes cause human sleeping sickness and Nagana in domestic animals. These parasites undergo part of their life cycle in mammalian hosts and part in their insect host, the tsetse fly, and are transmitted when the fly takes a blood meal. Within the fly, successful transmission involves two steps: colonisation of the midgut, and migration to the salivary glands. Although there are estimates of the number of trypanosomes that a fly can pick up from an infected host or transmit to a new host, it is not known how many parasites actually survive and contribute to an infection. We established a method to track parasites during transmission between individual mammals and flies, as well as in different host tissues. The same method can be applied to other parasites in a wide range of hosts and conditions. We found that a minimum of several hundred trypanosomes survived the transfer between hosts and contributed to a new infection. In contrast, parasites within a fly experienced a bottleneck during migration. An important outcome of this event is that even minor populations can be amplified in the glands of individual flies and disseminated, which could result in the rapid spread of new traits.

## Introduction

A bottleneck is an event in which the population size of a species is temporarily severely reduced. Bottlenecks can have strong evolutionary effects because limited population sizes can lead to dramatic shifts that favour certain genotypes (founder effects) [1], [2], [3] and to the stochastic loss of others [4], with rare genotypes being especially prone to being lost. Many digenetic parasites are presumed to experience bottlenecks because their population sizes are reduced during transmission between their two hosts, but there is little information on the size of such bottlenecks or the impact that this may have on genetic diversity. In addition, parasites may also encounter bottlenecks within a host as they differentiate and migrate from one tissue to another or infect different cell types.

**Figure 1:**
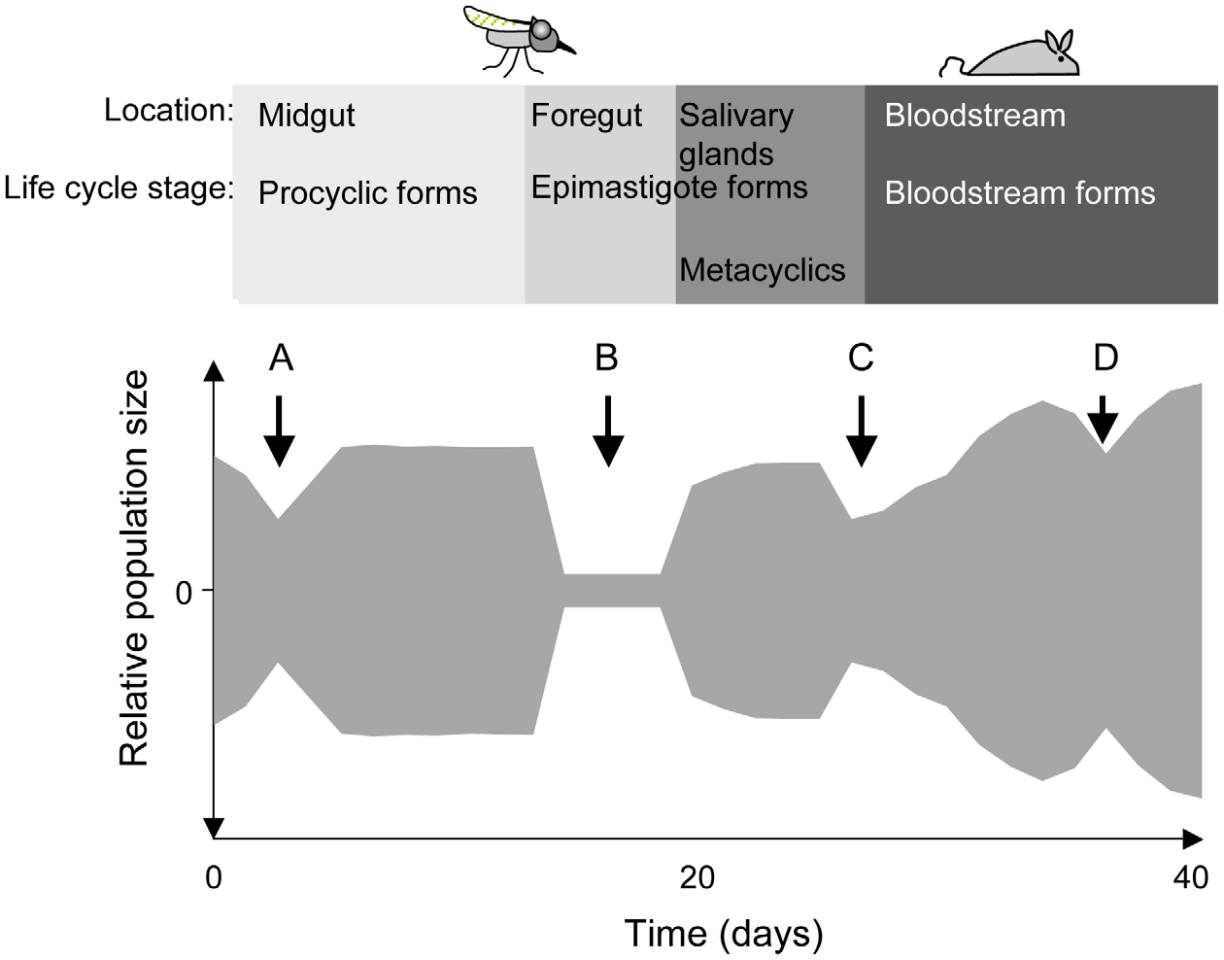
Schematic depiction of population bottlenecks during the life cycle of *Trypanosoma brucei*. The relative population size is shown on the y-axis. When trypanosomes are taken up by a tsetse fly, the population collapses during the adaptation to the midgut (A) and recovers thereafter. Only a few trypanosomes at a time are presumed to migrate to the salivary glands (B). Migration might take place during a defined period [6] or continuously [8]. Long epimastigote forms can reach the salivary glands where they deposit the short epimastigote forms that colonise the epithelia and give rise to metacyclic forms. During a blood meal, metacyclic forms are injected into a mammalian host. The injection and the relocation of trypanosomes from the site of injection into the bloodstream may reduce its number (C). In the mammalian host, the trypanosome population is periodically reduced in size owing to the adaptive immune response and to the differentiation of long slender bloodstream forms into non-dividing short stumpy forms (D).

The protozoan parasite *Trypanosoma brucei brucei* causes Nagana in cattle, while its close relatives *T. b. rhodesiense* and *T. b. gambiense* cause human sleeping sickness. All three sub-species undergo part of their developmental cycle in their insect vector, the tsetse fly (*Glossina spp.*), and part in their mammalian host. Within their life cycles, there are several phases where parasite numbers are severely reduced (shown schematically in Figure 1). When a fly feeds on an infected mammal, the parasites that are taken up reach the midgut together with the blood meal. Depending on the trypanosome density in the mammalian host, the fly may ingest anywhere from a few hundred to several hundred thousand organisms. Many species of tsetse are completely refractory to infection by a particular species of trypanosome (reviewed in [5]). Even when a fly species is susceptible, the number of parasites in the midgut can decrease by three orders of magnitude after 3–5 days [6] (Figure 1; A). Attrition of the parasite population also occurs when an infection is initiated with procyclic forms fed to flies through a silicon membrane [7] indicating that the drop in numbers is not solely due to parasites failing to differentiate. In many flies the infection is eradicated at this point; in flies that sustain an infection, the surviving parasites multiply as procyclic forms and colonise the ectoperitrophic space, reaching densities of up to 5×10^5^ parasites per midgut [6].

To complete the life cycle, trypanosomes must migrate to the salivary glands via the foregut and the proboscis [6], [8]. In a large proportion of tsetse flies with infected midguts, trypanosomes fail to infect the salivary glands [9], [10] (Figure 1; B). The factors that promote or hinder colonisation of the salivary glands are not known and it is under debate whether migration is continuous [8] or restricted to a defined period [6]. It has been proposed that only a few trypanosomes undertake this journey and that asymmetrically dividing epimastigotes are the only forms capable of colonising the salivary glands [6]. Two lines of evidence support the notion of a limited founder population in the glands: first, fewer than ten epimastigote forms could be detected in the salivary gland ducts of individual flies [6] and second, in mixed infections with two strains of trypanosomes, each tagged with a different fluorescent protein, colonisation of a gland by only one strain was observed on several occasions [11], [12].

Epimastigotes in the salivary glands attach to the epithelium and proliferate, giving rise to the mammalian-infective metacyclic forms [13] that are transmitted to a susceptible mammal during a blood meal. It has been estimated that flies can inject up to several thousand trypanosomes when they feed on a new host [14], [15], but it is not known how many of these differentiate into bloodstream forms and establish an infection (Figure 1; C). Within the mammalian host, a chronic infection is characterised by repeated waves of parasitaemia (Figure 1; D). These are due to the interplay between three phenomena: the host immune response to the parasite's variant surface glycoprotein (VSG) coat, resulting in elimination of the population that expresses this particular variant, outgrowth of minor populations that have switched to a different VSG, and differentiation of proliferating slender bloodstream forms to non-dividing stumpy forms at high parasite densities. Stumpy forms are preadapted for further differentiation in the fly and have a lifespan of only a few days in the mammalian bloodstream [16].

Given the right conditions, trypanosomes can infect their mammalian and insect hosts very efficiently: a single parasite is sufficient to infect a tsetse fly [17] and one bite of an infected fly is sufficient to infect a mammal [18] with a minimal infective dose of one metacyclic trypanosome [19]. This high infectivity implies that trypanosomes can cope with very narrow bottlenecks. If transmission bottlenecks are so small and so frequent, however, trypanosomes might risk a loss of fitness and the accumulation of deleterious mutations [20]. In addition, any acquired mutations (such as drug resistance) that are beneficial to the parasite in one host might be lost during transmission through the second host.

In the case of endoparasites, the quantification of bottlenecks can be difficult because populations are not easily observed over time. Furthermore, it is not straightforward to distinguish between random and selective population reduction. To resolve these problems we used a novel methodology to monitor the population dynamics of *T. b. brucei* in tsetse. This was subsequently extended to the rest of the life cycle, including transmission from the fly to the mammalian host and vice versa. Different strains of trypanosomes can vary greatly in their ability to be transmitted by tsetse [21], [22]. Genetic differences were minimised by tagging the progeny of a single trypanosome with short unique DNA sequences that were integrated into a non-transcribed region of the genome. These tags were subsequently used to identify the different populations by amplifying them by polymerase chain reaction and subjecting them to deep sequencing. This approach has the advantage that it yields quantitative data about the different populations that co-exist, as well as allowing an estimate of the population size after a bottleneck.

## Results

**Figure 2:**
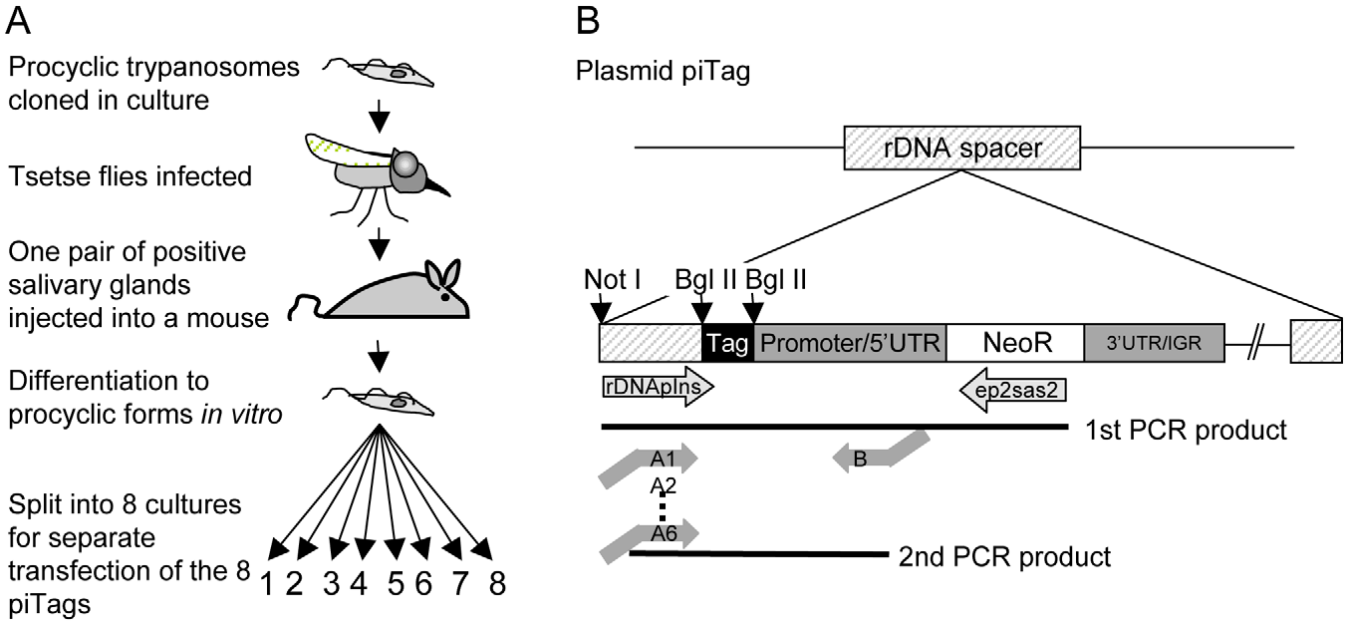
Cloning procedure and generation of tagged trypanosomes. A. Cloned procyclic trypanosomes were passaged through a tsetse fly and a mouse and bloodstream forms were triggered to differentiate to procyclic forms *in vitro*. The plasmids piTag 1–8 were then transfected separately into *T. brucei*. Cloned stable transformants containing each of the eight tags were isolated. B. Construction of the plasmid piTag. Eight different 40mers were integrated into the plasmid upstream of the procyclin promoter. Expression of the neomycin resistance gene (NeoR) in trypanosomes is controlled by the EP1 procyclin promoter and 5′ untranslated region (UTR) and the last 19 bases of the 3′ untranslated region and intergenic region (IGR) of EP2 procyclin. The linearised plasmid integrates into an rDNA spacer in the genome. Tags were amplified from genomic DNA by nested PCR.

Repeated syringe passage of bloodstream forms in rodents or prolonged culture of procyclic forms can reduce infectivity for flies. We therefore used the following protocol (Figure 2A) to obtain trypanosomes that were genetically homogeneous and capable of completing the life cycle: procyclic forms of *T. b. brucei* were cloned and a single clone was transmitted through a fly and a mouse. Bloodstream forms isolated from the mouse were triggered to differentiate to procyclic forms in culture. To generate parasites that were distinguishable from each other, aliquots of the culture were transfected with plasmids containing a unique 40bp tag (Figure 2B and Table S1). The tag in each plasmid lies upstream of the promoter and should not be transcribed, and therefore not influence the fitness of the parasite.

**Figure 3:**
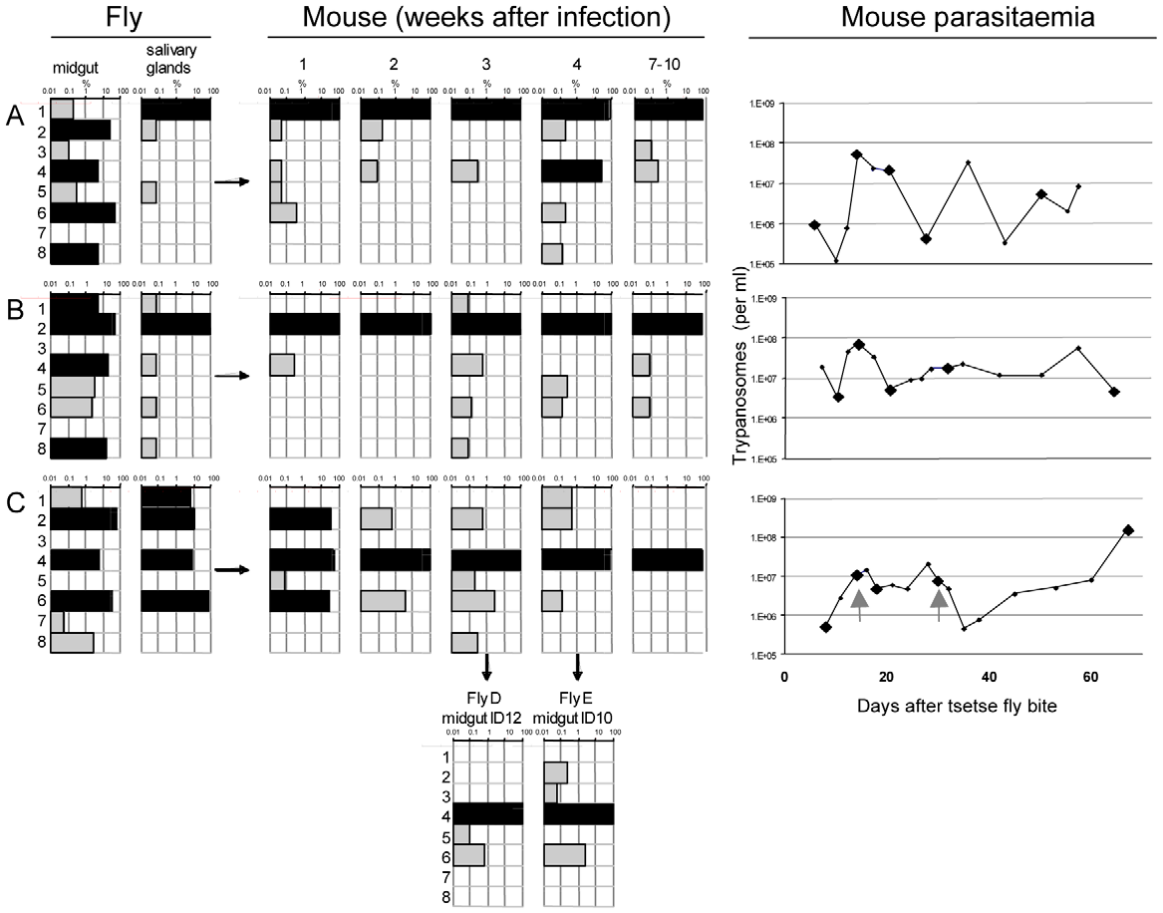
Diversity and frequency of tagged trypanosomes in three experiments. The bars in each chart represent the 8 tags. Frequency is shown on a log scale ranging from 0.01–100 per cent with dominant tags (>5%) highlighted in black. Tsetse flies were infected with procyclic trypanosomes with an even distribution of the tags. Three flies (A, B, and C) had infected midguts (mg) and salivary glands (sg). These flies each infected one mouse whose parasitaemia was monitored over a period of 2–3 months. Since the mice are immunocompetent, antigenic variation occurs, with each peak of parasitaemia containing trypanosomes expressing new VSGs compared to the preceding peaks. Five blood samples were taken from each mouse for analysis of the tags (indicated by large diamonds in the right panel) after 1, 2, 3, and 4 weeks and at the end of the experiment after 7–10 weeks. Flies D and E were infected on mouse C 18 and 30 days after infection (indicated with the grey arrows in the right panel). Midguts were dissected after 12 days (ID12) and 10 days (ID10). The number of sequences obtained from each sample is shown in Figure S2A.

Trypanosome clones (one for each of eight tags) were isolated and tested for growth in culture. All grew at similar rates (Figure S1). Cultures of the eight clones were mixed and used to infect tsetse. Three flies (A, B, and C) that were positive for metacyclic forms were selected and allowed to infect mice. Parasites were first detected in the corresponding mice 6, 7 and 4 days, respectively, after the infective bite. Subsequently, the salivary glands and midguts of the flies were isolated by dissection and DNA was extracted. Tail blood samples were collected from each mouse to monitor the parasitaemia and DNA was prepared from samples in weeks 1, 2, 3 and 4 and at the termination of the experiment after 7–10 weeks. Two batches of ten flies were fed on mouse C 18 and 30 days post infection. Midguts were dissected after 10 and 12 days and one positive midgut from each batch was taken for tag analysis (fly D and fly E in Figure 3). Tags were amplified by PCR, sequenced by 454 massively parallel pyrosequencing and analysed for their frequency and distribution. In total, we identified 30,592 sequences, an average of 1330 per sample (Figure S2). Control experiments confirmed that the barcoded primers did not affect the frequency with which individual sequences were detected (Figure S2).

A common pattern in all three experiments (Figure 3) was the large number of different tags detected in the midgut, many of them at high frequency (>5%). Each midgut contained at least 6 different tags and, taken together, all 8 tags could be detected in the three flies. This demonstrates that the procyclic culture forms used to infect the flies maintained their diversity in the gut lumen. The frequency of individual tags in the salivary glands changed compared to the midgut (Figure 3). This was most striking in fly A, in which tag 1 was minor (0.2%) and tag 6 dominant (52%) in the midgut, whereas in the salivary glands tag 1 was dominant (99.9%) and tag 6 undetectable. In fly B, four tags were dominant in the midgut, but only one of these (tag 2) was dominant in the salivary glands. In fly C, the three tags that were dominant in the midgut (tags 2, 4 and 6) were also dominant in the salivary glands, with tag 6 constituting 74% of the population. In addition, tag 1, which was present at <1% in the midgut, accounted for 6.9% of the parasites in the salivary glands. This analysis demonstrates that tags that are dominant in the midgut are not necessarily so in the salivary glands, and that their relative frequencies can be altered. This is reflected by the diverse correlation coefficients: r^2^ = 0.08, 0.86 and 0.25 for flies A, B, and C respectively, and implies that when trypanosomes are equally fit, any of the parasites from the midgut is capable of migrating to the salivary glands and founding the dominant population.

At the beginning of the infection in mice, the distribution and frequency of tags was very similar to the parasite populations in the salivary glands of the corresponding tsetse fly. The dominant tags in the salivary glands retained their dominance in mouse A and mouse B from the first sample onwards. In mouse C, tags 2 (32%), 4 (43%), and 6 (25%) were present in the first week of infection. Tag 4 was the only dominant tag from the second week onwards and finally the only one detectable after ten weeks. Tag 1, with a frequency of 6.9% in the salivary glands of fly C, became very minor in the following mouse infection and was detected only once after four weeks. The presence of one dominant tag and a few minor tags during mouse infections led to a very uneven distribution of individual tags, which showed up to a thousand-fold variation in frequency within a single sample. This uneven distribution is also reflected in a low Simpson's diversity index [23] during the course of infection in the mice (Table 1). Based on the parasite density and the frequency with which each tag occurred at the different time points sampled, the parasitaemia of individual populations could be extrapolated from the data (Figure S3). This revealed that the dominant and minor populations showed similar fluctuations in parasitaemia, although their titres differed by several orders of magnitude.

**Table 1:** Diversity index (Simpson's index).

	Fly		Mouse				
	midgut	sal. gland	week 1	week 2	week 3	week 4	week 7–10
Exp. A	0.595	0.002	0.009	0.005	0.006	0.399	0.007
Exp. B	0.660	0.006	0.006	0.000	0.018	0.008	0.003
Exp. C	0.550	0.428	0.652	0.088	0.079	0.023	0.000
Fly E	0.015						
Fly D	0.051						

Analysis of the tags present in the midguts of flies D and E, which became infected after feeding on mouse C, revealed two interesting outcomes. First, the tag that was dominant in the mouse remained so in the midgut of both flies (Figure 3). Second, minor tags in the bloodstream form population were also present. For example tags with frequencies of 0.5–3% in the bloodstream form population (tags 2, 5, and 6) were detectable in the midgut. Interestingly, tag 3 was detectable in fly E even though it was under the detection limit in all the samples from mouse C. Together, the two flies took up five different tags. This mouse had a parasitaemia of 4.9×10^6^ and 7.7×10^6^ per ml on days 18 and 30, respectively. Assuming the flies imbibed approximately 20µl of blood, about 1–1.5×10^5^ trypanosomes might reach the midgut of each fly. If approximately 1% survived [6] even very minor tags would be represented by 2–30 individual trypanosomes that could contribute to establishing the midgut infection.

## Discussion

By using tagged trypanosomes originating from a single clone, we have been able to monitor the dynamics of a parasite population throughout the life cycle without the confounding factor of strain differences. This analysis revealed that a major bottleneck in the life cycle occurs during migration of parasites from the midgut to the salivary glands, leading to the establishment of one or a few dominant genotypes in each fly. Minor genotypes constituting <1% of the population could also be detected in the glands, however. These were transmitted to mice together with the dominant genotype(s), and were found to persist during a chronic infection and survive transmission to a new insect vector.

**Figure 4:**
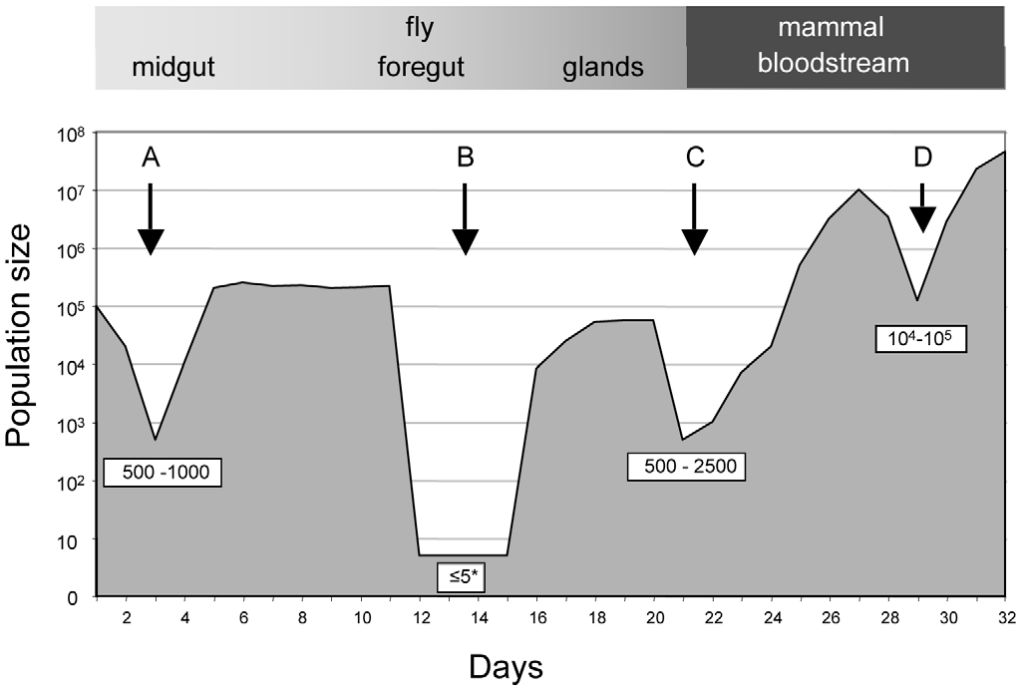
Population size through the life cycle of *T. brucei* based on data from this study. A: establishment of infection in the fly midgut following a blood meal; B: trypanosomes migrating to the salivary glands; C: establishment of infection in the mammal following fly transmission; D: fluctuations in parasite numbers in the mammalian bloodstream. Numbers in boxes refer to minimum numbers of trypanosomes surviving the transition between different tissues and hosts. The asterisk depicts the possibility that there might be waves of migration of a few parasites at a time from the midgut to the salivary glands.

The frequency and diversity of tags enabled us to extrapolate the minimum number of parasites transferred between hosts and provide an estimate of the size of the bottleneck (Figure 4). We estimate that at least 500–1000 trypanosomes must have survived the transfer from mouse C to flies D and E and colonised their midguts. The population structure was similar to that in the mouse blood at the time of the blood meal, indicating that despite the reduction in parasite numbers, transmission from the mammal to the insect does not represent a severe bottleneck.

Likewise, the minimum number of trypanosomes that survived the transfer from infected flies and initiated the infection in the 3 mice was estimated by drawing simulation to range from 500–2500 (Figures 4 and S4). This is similar to the number of metacyclic forms extruded by infected flies [14], [15], implying that most of the parasites that are inoculated can contribute to an infection. With the exception of a single major tag that was present in the salivary glands of fly C, parasites isolated from mice during the first week of infection reflected the diversity and distribution of parasites in salivary glands of the infecting fly. A large number of tags were detected during the chronic infections, but their frequency was highly variable. Some tags were detected only once in a total of 6800 sequences obtained from five blood samples (e.g. tag 1 or tag 8 in mouse B). Considering that the parasitaemia never fell below 10^5^ ml^−1^, we consider it likely that most tags transmitted by the tsetse were present continuously during mouse infection although they were under the detection limit in individual samples. Antigenic variation must have occurred several times during the course of these infections, since the mice were immunocompetent. Ordered (hierarchical) VSG expression is a widely accepted model for antigenic variation [24], [25], [26]. Since the tagged trypanosomes derive from a clone, it is possible that they obeyed the same hierarchy, which would result in synchronised VSG expression by trypanosomes with different tags. This is not mandatory, however, as simultaneous expression of several variants is both predicted by the model of Lythgoe et al. [26] and can occur in vivo [27].

In contrast to transmission between hosts, migration from the midgut to the salivary glands of tsetse caused profound changes in relative frequencies of different tags, with up to five tags per experiment changing from dominant to minor, or vice versa. Two flies had a single dominant tag accounting for more than 99% of the population in the glands, while the remaining fly had 4 dominant tags (one of which accounted for 74% of the population). When minor populations from the glands and the corresponding mice were taken into account, all the tags that were found in the midgut were represented, meaning that at least 6 trypanosomes must have reached the salivary glands in each fly. The strong dominance of one tag each in flies A and B, and to a slightly lesser extent in Fly C, could best be explained by waves of migration by very few parasites at a time. Trypanosomes are tightly packed in infected glands and have to compete for space. Early migration might be a more important factor than dominance in the midgut if parasites that arrive first can disperse and colonise the glands more readily than latecomers. This “race for space” would account for the shift in frequencies between the midgut and salivary glands of a single fly and also explain why a single tag can dominate the population while others remain very minor. It would also be compatible with publications that have reported changes in the relative frequencies of two strains of trypanosomes between the midgut and glands, as well as glands colonised by only one of the two strains [11], [12]. The latter study catalogued whether each of the pair of salivary glands contained one or both strains, thus allowing the minimum number of founder trypanosomes in each fly to be extrapolated from the data. In these experiments the salivary gland infections of approximately two-thirds of the flies could have been established by as few as one or two trypanosomes, while the remaining third would have required at least three or four. It could not be excluded, however, that several trypanosomes of one strain migrated to the same gland, or that very minor populations might have been overlooked.

An important outcome of the extreme bottleneck that can occur between the midgut and the salivary glands is that rare variants can be amplified in individual flies and be disseminated by them. If a variant has a selective advantage in mammals, such as altered host range or increased resistance to drugs, this might cause it to become the major species circulating locally [28], [29]. Such a phenomenon could explain the epidemic population structure of *T. brucei* documented by MacLeod and coworkers, in which clonal expansion of a few genotypes in a region occurs against a background of frequent recombination between strains [30].

Our data indicate that both mammals and tsetse can readily acquire and transmit more than one genotype in the course of a single blood meal. Mixed genotypes have been detected fairly frequently in field isolates from cattle, humans and tsetse [30], [31], [32], [33], and it is possible that they might be even more widespread since minor populations would escape detection. Co-infection of susceptible flies with different strains of trypanosomes might affect parasite population dynamics [34] and also increase the chances of genetic exchange [35], [36] if the parasites develop and migrate in parallel.

The approach that we have used here can be extended to study other facets of infection with trypanosomes, for example the population found in the central nervous system during the late stage of sleeping sickness or the parasites causing relapse infections after drug treatment. It can also be applied to the analysis of population dynamics of any other parasite that is amenable to transfection, including other African trypanosomes that do not infect the salivary glands and may therefore show different dynamics in the tsetse fly.

## Materials and Methods

### Ethics statement

Animal experiments were approved by the local veterinary authorities (Veterinäramt Basel-Stadt) in compliance with Swiss federal law (TSchG) and cantonal by-laws (TSchV Basel-Stadt).

### Trypanosomes

*Trypanosoma brucei brucei* AnTat 1.1 [37] procyclic forms were cloned by the micro-drop method [38] and stabilates were made after 22 days. Tsetse flies (see below) were infected with one clone, and the infected salivary glands of one fly were dissected at day 27 and inoculated intraperitoneally into a female NMRI mouse (RCC, Ittingen, Switzerland), which developed a parasitaemia of 10^8^ trypanosomes ml^−1^ at day 5 post infection. Bloodstream forms, obtained by heart puncture, were triggered to differentiate into procyclic forms in SDM-79 supplemented with 10% foetal bovine serum, 3 mM sodium citrate and cis-aconitate (CCA) [39], and 20mM glycerol at 27°C for 3 days [40]. Procyclic forms were cultured thereafter in the same medium without CCA.

### Infection of tsetse flies

Pupae of *Glossina morsitans morsitans* were obtained from the Institute of Zoology, Bratislava, Slovakia. The flies were maintained at 25°C and 70% relative humidity with 12 hours of light per day. Teneral tsetse flies (under the age of 72 hours) were infected with procyclic forms as described previously [22]. Starting twenty days post-infection, tsetse flies were examined for the presence of metacyclic forms in their saliva. Tsetse flies with a mature infection were allowed to feed on NMRI mice 2 to 4 days after the appearance of first metacyclic forms. Subsequently the paired ducts of the salivary glands were extracted from the neck of the tsetse flies. This prevented contamination with midgut forms. The midgut (including the proventriculus) was then dissected out of the abdomen. The tissues were dissected on separate slides in a drop of PBS and then transferred to an Eppendorf tube containing 200µl lysis buffer (see below) and stored at −20°C prior to DNA extraction.

### Infection of mice

Female NMRI mice (RCC, Ittingen) were kept at 22°C, 70% relative humidity and with 12 hours of light per day. To determine the parasitaemia, 10 µl tail blood were mixed with 40 µl 3.2% sodium citrate, and 4 µl were uniformly distributed under a 20 mm^2^ cover slip. For each sample, 10–15 fields were counted. The parasitaemia is given as the number of trypanosomes per ml mouse blood. For analysis of the tags 50 µl of tail blood was processed.

### Plasmid constructs and stable transfection of trypanosomes

The insert from the plasmid pKON [21], was amplified by PCR using the primers Bgl II-promotor (ATAGATCTCGAAAACTCTTCGGGA) and KO 2 (TATCTAGAGGGCACTGCAGT). Bgl II and Xba I sites, respectively, are underlined. The PCR product encompassing the EP1 promoter, the neomycin resistance gene and 19bp of the EP2 3′ untranslated region (UTR) was digested with Bgl II and Xba I. The plasmid pLew111 (http://tryps.rockefeller.edu/) was digested with Bgl II and Nhe I to provide the plasmid backbone with the rDNA spacer. The digested PCR product was ligated to the backbone (Xba I and Nhe I have compatible ends). The resulting construct, pIns has a single Bgl II site between the rDNA spacer and the procyclin promoter that was used for the insertion of unique tags (Figure 2B).

An oligonucleotide (ATCACGGCCGGGAGATCT (N)_40_ AGATCTGTGAGACCCATTAAGCTTCC) containing a variable 40mer flanked by two constant regions with Bgl II sites (underlined), was purchased from Microsynth AG, Balgach, Switzerland. Double-stranded DNA was produced by amplification with the constant flanking sequences: iTag-oligo (ATCACGGCCGGGAGATCT ) and BIL-4A (GGAAGCTTAATGGGTCTCAC ). The PCR product was inserted into pCR2.1 TOPO (Invitrogen, Carlsbad Ca, USA) according to the manufacturer's protocol and used to transform *E. coli* XL-1 blue. Purified plasmids were sequenced using standard methods. Eight tags were selected (Table S1a), the fragments released with Bgl II and ligated into pIns to generate the plasmid series piTag1–8.

Transfection was performed with 10µg of each plasmid (piTag1–8) linearised with Not I. Plasmids were electroporated separately into 2.5×10^7^ procyclic trypanosomes. Transfection and cloning by limiting dilution were carried out as described elsewhere [40]. G418 (25µg ml^−1^) was used to select stable transformants.

### DNA extraction, nested PCR and sequencing

Samples were resuspended in 200µl lysis buffer (100 mM NaCl, 5 mM sodium EDTA, 10 mM tris-HCl, pH 8), supplemented with 20 µl RNAse A (1 mg/ml) and incubated for 1 h at 37°C, followed by the addition of 10µl Pronase (20mg/ml) and incubation for a further 2 h. Genomic DNA was isolated by phenol/chloroform extraction, precipitated with ethanol and resuspended in 50 µl water. Genomic DNA obtained from blood samples was subjected to an additional precipitation using 0.5 volumes of 7.5M ammonium acetate and 5 volumes ethanol.

Nested PCR was performed on 1 µl of each DNA sample. The oligonucleotides rDNAsense/ep2sas2 (Table S1b) were used to generate a product of ∼700 bp using the following conditions: 3 min at 96°C, 30 cycles of 1 min 94°C, 1 min 45°C, and 45 sec 72°C, followed by 10 min extension at 72°C. The second PCR performed with 1 µl from the first reaction as template and the fusion primers A and B, these consist of two regions: a template specific region for PCR amplification and a fusion region for 454 sequencing (Microsynth, Balgach, Switzerland). The primers A1–A6 can be distinguished by a variable 6mer barcode that connects the two regions (Table S1b). This barcode was used to allocate samples in the same region on the pyrosequencing plate (see below). The PCR conditions with the primers A and B were: 3 min at 96°C, 30 cycles of 1 min 94°C, 50 sec 52°C and 30 sec 72°C followed by 10 min extension at 72°C, yielding products of 177 bp. 454 picotiter plate pyrosequencing was performed by Microsynth, Balgach, Switzerland, with the Roche Genome Sequencer FLX System as described elsewhere [41], [42]. Five regions of a picotiter plate were used for this study: one (I) for the control DNA and four regions (II–V) for the samples (Figure S2, panel A). For the control sequencing reactions, cultures of individual clones were mixed, genomic DNA extracted, and split into 6 aliquots. DNA from each aliquot was amplified with a different barcoded fusion primer A1–A6 together with the primer B. The samples collected from the flies and mice were amplified with the primers A1/B–A6/B as indicated in Figure 2. The barcodes were identified with the SFF file program sfffile (included in the 454 software package), allowing one mismatch. The distribution of the tags (Figure S2, panel B) was very similar among all control samples (two-sided paired T-test; p>0.3 for all combinations).

## Supporting Information

Figure S1 Growth of tagged procyclic forms in culture. Each colour corresponds to a clone carrying one of the tags.
(0.02 MB PDF)

Figure S2 A: Organisation of samples on the pyrosequencing plate. Each row represents one region (I–V) where six samples were mixed, each amplified with a forward primer with a different barcode (A1–6). The number of sequences obtained for each sample is given. B: A control culture containing a mixture of all 8 tags was amplified with each of the barcoded primers. The distribution was very similar, indicating that the different barcodes did not bias the analysis.
(0.06 MB PDF)

Figure S3 Parasitaemia of each ‘tag population’ in the three mouse experiments.
(0.06 MB PDF)

Figure S4 Drawing simulation of the minimum number of trypanosomes transmitted from tsetse flies to the three mice. Different numbers of individuals were randomly drawn (with replacement) 10000 times from the distribution of tags in the salivary glands and the number of different tags drawn recorded each time. Tags not observed in the salivary glands but recorded in the subsequent mouse or fly samples were added at half the frequency of tags found in only one sequence in the salivary gland. The number of individuals below which one or more tags would be lost in 95% of cases was taken as the most conservative estimate of the minimum bottleneck size. A, B and C correspond to the transmission in the respective experiments (see Fig. 3).
(0.06 MB PDF)

Table S1 Tags and primers used in this study A. Sequences of Tags 1–8. B. Sequence of primers used for nested PCR. The primers A1–A6 and B consist of a fusion region (in italics on the left) and a template specific region (on the right). The variable 6mer barcode of the primers A1–A6 is underlined. The barcode allows the allocation of samples in the same region on the pyrosequencing plate.
(0.08 MB PDF)
