# Supplementary figures and images for: Bottlenecks and the Maintenance of Minor Genotypes during the Life Cycle of Trypanosoma brucei
Source: PLoS Pathog. 2010 Jul 29;6(7):e1001023. doi: 10.1371/journal.ppat.1001023 (PMC2912391; doi:10.1371/journal.ppat.1001023)

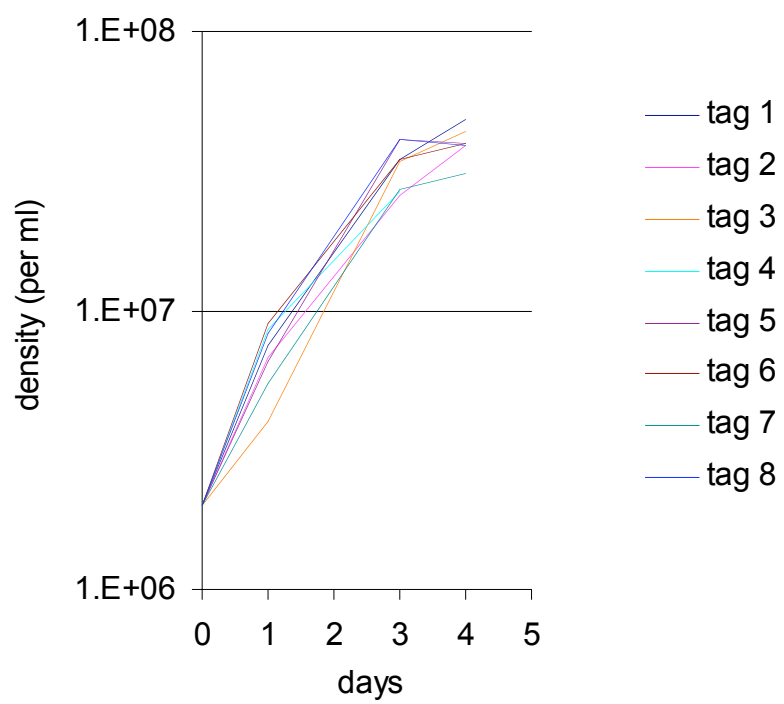

Supplement: Figure S1 — Growth of tagged procyclic forms in culture. Each colour corresponds to a clone carrying one of the tags. (0.02 MB PDF) [file ppat.1001023.s001.pdf]

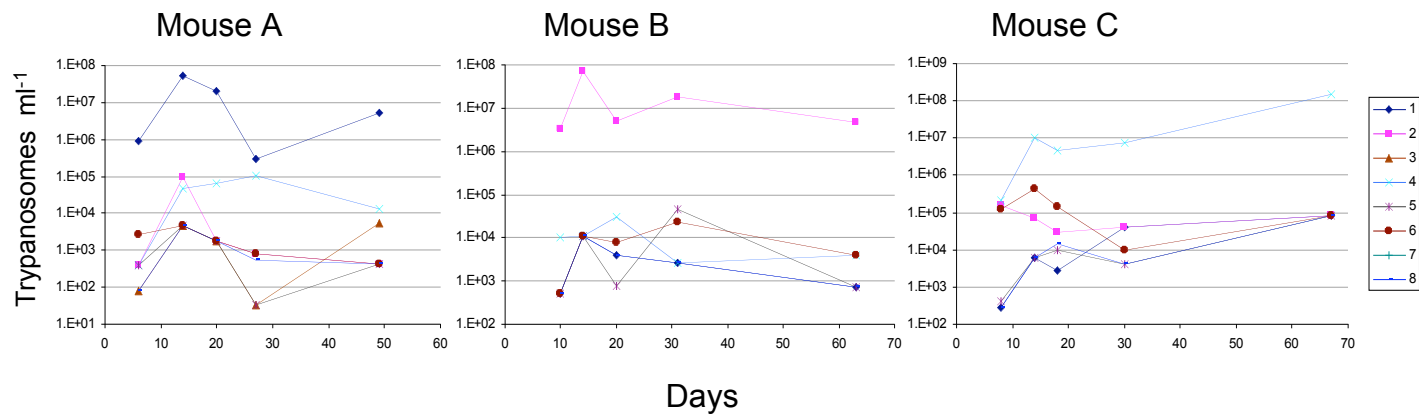

Supplement: Figure S3 — Parasitaemia of each ‘tag population’ in the three mouse experiments. (0.06 MB PDF) [file ppat.1001023.s003.pdf]

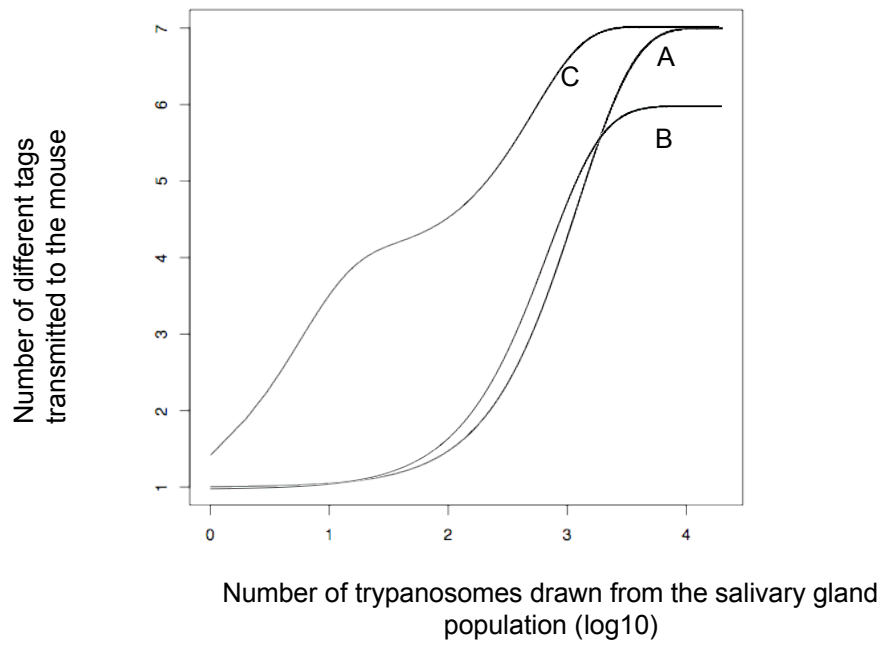

Supplement: Figure S4 — Drawing simulation of the minimum number of trypanosomes transmitted from tsetse flies to the three mice. Different numbers of individuals were randomly drawn (with replacement) 10000 times from the distribution of tags in the salivary glands and the number of different tags drawn recorded each time. Tags not observed in the salivary glands but recorded in the subsequent mouse or fly samples were added at half the frequency of tags found in only one sequence in the salivary gland. The number of individuals below which one or more tags would be lost in 95% of cases was taken as the most conservative estimate of the minimum bottleneck size. A, B and C correspond to the transmission in the respective experiments (see Fig. 3). (0.06 MB PDF) [file ppat.1001023.s004.pdf]
