# Supplementary material for: Bottlenecks and the Maintenance of Minor Genotypes during the Life Cycle of Trypanosoma brucei
Source: PLoS Pathog. 2010 Jul 29;6(7):e1001023. doi: 10.1371/journal.ppat.1001023 (PMC2912391; doi:10.1371/journal.ppat.1001023)

A

Oligonucleotide used	region I	region II	region III	region IV	region V
**A1**	control sample A1 1447seq.	FlyA sg 1611 seq.	FlyA midgut 980 seq.	mouseA ID 6 2340 seq.	mouseA ID 14 1167 seq.
**A2**	control sample A2 1605 seq.	mouseA ID 20 1257 seq.	mouseA ID 27 1514 seq.	mouseA ID 49 1908 seq.	FlyB sg 1266 seq.
**A3**	control sample A3 1052 seq.	FlyB midgut 993 seq.	mouseB ID10 1035 seq.	mouseB ID 14 1828 seq.	mouseB ID 20 1314 seq.
**A4**	control sample A4 1214 seq.	mouseB ID 31 1535 seq.	mouseB ID 63 1186 seq.	FlyC sg 540 seq.	FlyC midgut 792 seq.
**A5**	control sample A5 1125 seq.	mouseC ID 8 1248 seq.	mouseC ID 14 1180 seq.	mouseC ID 18 1033 seq.	mouseC ID 30 1794 seq.
**A6**	control sample A6 1476 seq.	mouseC ID 67 1366 seq.	FlyD midgut ID12 1070 seq.	FlyE midgut ID10 1635 seq.	
seq. per region	7919	8010	6965	9284	6333
total Sequences					38511

B

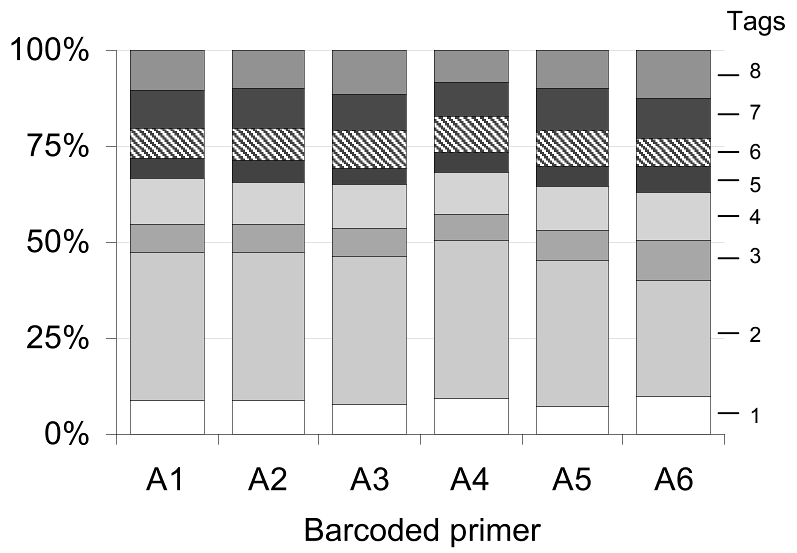

Supplement: Figure S2 — A: Organisation of samples on the pyrosequencing plate. Each row represents one region (I–V) where six samples were mixed, each amplified with a forward primer with a different barcode (A1–6). The number of sequences obtained for each sample is given. B: A control culture containing a mixture of all 8 tags was amplified with each of the barcoded primers. The distribution was very similar, indicating that the different barcodes did not bias the analysis. (0.06 MB PDF) [file ppat.1001023.s002.pdf]
