# Supplementary material for: Bottlenecks and the Maintenance of Minor Genotypes during the Life Cycle of Trypanosoma brucei
Source: PLoS Pathog. 2010 Jul 29;6(7):e1001023. doi: 10.1371/journal.ppat.1001023 (PMC2912391; doi:10.1371/journal.ppat.1001023)
Supplement: Table S1 — Tags and primers used in this study A. Sequences of Tags 1–8. B. Sequence of primers used for nested PCR. The primers A1–A6 and B consist of a fusion region (in italics on the left) and a template specific region (on the right). The variable 6mer barcode of the primers A1–A6 is underlined. The barcode allows the allocation of samples in the same region on the pyrosequencing plate. (0.08 MB PDF) [file ppat.1001023.s005.pdf]

Table S1

A. Sequences of Tags 1-8

	Sequence 5' - 3'
**Tag 1**	GGCGGTGGTGCCCTGTTCCT AAGAGTTTTTTTGCGATGAG
**Tag 2**	TTGGGCTAGCGTTCGTGTTC GCTTGCTATTGTTATTCTTT
**Tag 3**	ATTCTTGCCTAATGTGCGAG CGTCTAGCTGGATGGGGTTT T
**Tag 4**	CACAAGCGTGGATAACCGGA TTACGTCTTTCGGTGTGTCC
**Tag 5**	GCCTTTCTCGCGTGGACATG CTTTTAAGGTATGCACCGAG
**Tag 6**	GTGGGGCGCTTAGGTTCTGA TATGGGACGGTGAAATCGCA
**Tag 7**	TGTATTCCATGCCTATCCTC AGGGGAATGGCAGTGTAACG
**Tag 8**	TGATTTAATTGTTGAAAGAG TGATTGGGTTCATATTTCGC

B. Sequence of primers used for nested PCR. The primers A1 - A6 and B consist of a fusion region (in italics on the left) and a template specific region (on the right). The variable 6mer barcode of the primers A1 - A6 is underlined. The barcode allows the allocation of samples in the same region on the pyrosequencing plate.

Oligonucleotide	Sequence, 5' - 3'
rDNApIns	GAGGACCGAATACTAATA
ep2sas2	TATAGATCTGTGAATTTTACTTTTTGGT
Fusion primers	
A 1	*GCCTCCCTCGCGCCATCAG* <u>TACGAG</u> AGAATGTCTTTGGCAACACAC
A 2	*GCCTCCCTCGCGCCATCAG* <u>ATACGT</u> AGAATGTCTTTGGCAACACAC
A 3	*GCCTCCCTCGCGCCATCAG* <u>CGTATC</u> AGAATGTCTTTGGCAACACAC
A 4	*GCCTCCCTCGCGCCATCAG* <u>TCGACA</u> AGAATGTCTTTGGCAACACAC
A 5	*GCCTCCCTCGCGCCATCAG* <u>ACAGTG</u> AGAATGTCTTTGGCAACACAC
A 6	*GCCTCCCTCGCGCCATCAG* <u>CATGAC</u> AGAATGTCTTTGGCAACACAC
B	*GCCTTGCCAGCCCGCTCAG* TTCCGTGGGCCCCGACT
